# The Epigenetics of Anxiety Pathophysiology: A DNA Methylation and Histone Modification Focused Review

**DOI:** 10.1523/ENEURO.0109-21.2021

**Published:** 2023-04-21

**Authors:** Nikita S. Persaud, Hannah M. Cates

**Affiliations:** Biology Department, Adelphi University, Garden City, New York 11530

**Keywords:** anxiety disorders, DNA methylation, histone modifications, neuroepigenetics, review, rodent models

## Abstract

Anxiety is one of the most common psychiatric disorders diagnosed in the United States today. Like all mental illnesses, anxiety pathology includes genetic, molecular, somatic, and behavioral characteristics. Specific brain regions implicated in anxiety include the prefrontal cortex, amygdala, hippocampus, and hypothalamus. Together, these regions regulate fear-related learning and memory processes, and are innervated by neuronal projections that use glutamate and GABA as neurotransmitters. Neurotrophic factors such as brain-derived neurotrophic factor (BDNF) are also implicated in anxiety. This review discusses the neuroepigenetics of the anxiety phenotype. While studying such changes is limited to postmortem brain studies or peripheral tissue acquisition in humans, the use of animals to model anxiety phenotypes has made epigenetic research possible. In this review, we summarize and discuss a plethora of DNA methylation, histone modification, and associated gene expression differences underscoring the anxiety phenotype. The findings we outline include expression changes of various DNA methyltransferases and changes in histone modifications that affect the hypothalamic pituitary adrenal axis and stress response as well as GABA, glutamate, and BDNF signaling in the PFC, amygdala, hypothalamus, and hippocampus. Furthermore, there have been studies showing that anxiety behaviors and biological scars from stress can be reversed using histone deacetylase inhibitors, and we discuss ideas for the future of treatment. In this review, we hope that by compiling much of the data pertaining to DNA methylation and histone modifications *in vivo* animal studies we are able to highlight potential avenues for future research despite existing limitations.

## Introduction

Coined in 1942 by Conrad Waddington, epigenetics, from the Greek “epi” meaning over or above, genetics, is the accepted link between the ability of the environment to influence the genome of an organism to alter gene expression and, thus, the observed phenotype (Tronick and Hunter, 2016). Epigenetics therefore accounts for the way environmental factors like stress, “speak” to genes to modulate gene expression. In neuroepigenetics, these modifications alter brain plasticity, and may render an individual predisposed toward developing mental illnesses such as anxiety disorders (ADs; Schiele and Domschke, 2018). Epigenetic modifications do not change one’s DNA sequence; rather, they modulate levels of gene expression via the following four main mechanisms: DNA methylation, histone modifications, noncoding RNA interactions, and nucleosome positioning (Schiele and Domschke, 2018). In this review, the more prominently studied of these mechanisms in anxiety research, DNA methylation and histone modifications, are discussed in detail.

### DNA methylation

DNA methylation involves the covalent transfer of a methyl group from *S*-adenyl methionine to the C-5 position of a cytosine ring of DNA to form 5-methylcytosine (5mC; Moore et al., 2013). This reaction is catalyzed by a large class of enzymes known as DNA methyltransferases (DNMTs), composed of DNMT1, DNMT2, DNMT3A, DNMT3B, and DNMT3L (Morris et al., 2016). Additionally, DNA methylation is mediated by a family of proteins that bind 5mC, including methyl-CpG-binding protein (MeCP2; Martinowich et al., 2003). Oftentimes, when methylation marks are present on gene promoters, usually in CpG islands, gene transcription is repressed (Jin et al., 2011).

### Histone modifications

In eukaryotic organisms, DNA is compacted into basic repeating units called nucleosomes, which are composed of an octamer of the four core histone protein subunits: H2A, H2B, H3, and H4 (Grant, 2001). DNA is coiled around these histone proteins to form a nucleosome, and, in turn, these nucleosomes are packed together to form chromatin. Each core histone carries a tail that extends into the space surrounding a nucleosome, providing sites for a wide variety of post-translational modifications. These modifications or markers include methylation, acetylation and phosphorylation, to name a few, and are catalyzed by specific enzymes (Grant, 2001). Histone markers interact with other proteins present in the nucleus to form complexes that shift the structure of chromatin at specific sites along the genome, between heterochromatin (compactly packed, repressed transcription) and euchromatin (loosely packed, increased transcription; Sun et al., 2013).

The acetylation state of histones is regulated by the following two classes of enzymes: histone acetyltransferases (HATs), which add acetyl groups to histone tails at lysine residues, and histone deacetylases (HDACs), which removes these acetyl groups. There are two types of HATs, type A and type B, with type A being divided into the following three main families of HATs: Gcn5-related *N*-acetyltransferases, MYST (named for a collection of genes), and CREB-binding protein (CBP/p300). (Bannister and Kouzarides, 2011). There are four classes of HDACs (classes 1–4), with HDACs of classes 1 and 2 assuming more major roles in the nervous system (Abel and Zukin, 2008). Class 1 consists of HDAC1 and HDAC2, while class 2 is composed of HDAC4, HDAC5, and HDAC7. Most often, acetylated histones are associated with transcriptionally active chromatin, as it allows increased access of transcription factors to exposed gene promoters and transcription start sites (TSSs), while deacetylated histones are associated with inactive transcription, though there are repressive acetyl marks (de Ruijter et al., 2003).

Histone methylation is regulated by histone methyltransferases (HMTs), which may add multiple methyl groups at lysine or arginine residues. One can therefore find histone tails that are monomethylated, dimethylated, or trimethylated (Grant, 2001). Histone lysine methyltransferases methylate histones at lysine residues, while protein arginine methyltransferases methylate histones at arginine residues (Bannister and Kouzarides, 2011). Histone methylation can be either repressive or permissive, depending on the location and number of methyl groups, as we will see in the studies discussed later.

### AD pathology

AD pathogenesis is multifactorial: development of an anxiety disorder involves biological, environmental, and psychological factors. Early-life stressors (prenatal or postnatal), substance abuse in adolescence and adulthood, as well as genetics influence one’s risk for developing an AD, though it is understood that these factors do not entirely account for AD pathogenesis, and may also contribute to the development of other disorders such as mood and depressive pathologies (Schiele and Domschke, 2018). Thus, there has been an increased focus on identifying factors that contribute to an individual’s resiliency or susceptibility toward developing an AD. In this review, we focus on summarizing differential DNA methylation and histone modification findings in anxiety models compared with control counterparts. Note that when studying anxiety in animal models, stress response is used as a proxy to study anxiety response.

When we consider the pathways involved in an anxiety-conditioned or fear-conditioned response, the question arises: what factors influence memory formation, stimulus conditioning, and subsequent anxious behavior learned? The pathophysiology of AD demands that specific brain regions, their associated neurotransmitters, the hypothalamic–pituitary–adrenal (HPA) axis, and its hormone components, all be considered when investigating anxiety from a genetic and epigenetic point of view (Bartlett et al., 2017). In humans, the brain is implicated in all psychiatric disorders. It is particularly responsive to stress and has the capacity for reversible structural changes (plasticity) that enables us to continuously adapt to the changing environment. Stress has been shown to induce such changes in various limbic system structures, including the amygdala and the hippocampus. The amygdala is actively engaged in ambiguous situations and contributes to the shaping of perception and value representation, such as labeling an object or experience as “rewarding” or “aversive.” The hippocampus facilitates contextual fear learning, which underlies the anxiety phenotype (Pessoa, 2010; Zhang et al., 2014). These neuronal changes include dendritic remodeling (expansion and contraction of dendritic trees), turnover of synaptic connections, and limited neuronal replacement via neurogenesis. Resilience associated with plasticity is often lost with age, resulting in impaired reversibility of these changes (Hunter and McEwen, 2013).

In the CNS, this plasticity is modulated by neurotrophic factors, which promote neuronal growth, survival, and regeneration, and are commonly referred to as neurotrophins (Xiao and Le, 2016). One such example is brain-derived neurotrophic factor (BDNF), which is a small protein encoded by the *BDNF* gene essential for neuronal growth, differentiation, and the overall development of the CNS. This includes brain repair following injury and the formation of long-term memory, such as the consolidation of aversive memories or constructive learning, all of which may contribute to the development of an AD (Mitte, 2008; Cattaneo et al., 2016).

Additionally, brain regions implicated in fear and anxiety responses are interconnected and capable of communicating with each other via the action of neurohormones and neurotransmitters, all of which are potential targets for anxiety-based epigenetic investigations. GABA is the major inhibitory neurotransmitter in the mature mammalian brain and CNS. It is capable of binding to two main receptors, GABA_A_ and GABA_B_, which are gated Cl^–^ channels (Valenzuela et al., 2011). On average, approximately one-third of CNS neurons use GABA as a neurotransmitter, particularly interneurons. GABAergic neurons from the central amygdala (CeA), an integration center that converts emotionally relevant sensory information into physiological and behavioral responses, project into the hypothalamus (Gilpin et al., 2015). These projections dampen hypothalamic activity, such as autonomic anxiety responses to fearful stimuli (Gilpin et al., 2015; Nuss, 2015). Decreased GABA activity is often anxiogenic; that is, it induces anxiety (Nuss, 2015). Studies conducted in animals have shown that the administration of GABA receptor agonists into the amygdala leads to a decrease in fear and anxiety observed in these models (Nuss, 2015). Benzodiazepines, a class of antianxiety medication, enhance the neuronal inhibitory action of GABA via allosteric effects at GABA receptors, leading to enhanced anxiolytic (reduced anxiety) or tranquilized states (Bleakley and Davies, 2014). Thus, several studies discussed later investigate differential DNA methylation or histone modifications associated with genes associated with GABA and its receptors.

The amino acid glutamate is the major excitatory neurotransmitter in the mammalian brain and CNS that counterbalances the inhibitory actions of GABA (Nuss, 2015). This cooperation can be seen when considering the previously mentioned GABAergic pathway starting in the CeA. Before the CeA can exert its inhibitory effect on the hypothalamus, it receives glutamatergic or excitatory input from the basolateral amygdala (BLA), which has been implicated in pavlovian learning and receives input from the parietal, cingulate, and prefrontal cortices (Pessoa, 2010; Nuss, 2015). In addition to its role at synapses, glutamate is also partially responsible for neurogenesis, synaptogenesis, and neurite outgrowth, similar to the neurotrophin BDNF. It is capable of binding to two types of receptors, NMDA and AMPA, which are gated Ca^2+^ and Na^+^/K^+^ channels, respectively (Riaza Bermudo-Soriano et al., 2012). It has been previously demonstrated that the inhibition of NMDA receptors at synapses blocks fear acquisition, and that acute stress appears to increase glutamate release as well as glutamate receptor expression, particularly the NMDA receptors that modulate the secretion of corticotropin-releasing factor (CRF)/corticotropin-releasing hormone (CRH), discussed later, in the CeA (Levenson and Sweatt, 2005; Riaza Bermudo-Soriano et al., 2012). Many antagonists of NMDARs and AMPARs in animal models have shown anxiolytic (reduced anxiety) outcomes, suggesting that these receptors may be possible pharmacological targets for treating ADs (Riaza Bermudo-Soriano et al., 2012).

Hormonal activity also plays a pivotal role in the anxiety response. The most well studied governor of the stress response, the HPA axis, involves a negative feedback mechanism among the hypothalamus, the pituitary gland, and the adrenal glands (Klengel et al., 2014). The paraventricular nucleus (PVN) of the hypothalamus secretes arginine vasopressin and CRF into the hypophyseal portal system, a capillary bed that connects the hypothalamus to the anterior pituitary gland (Herman et al., 2016). Here, these neuroendocrine chemicals promote the production of adrenocorticotropic hormone (ACTH), which is released into the bloodstream. ACTH then binds to ACTH or melanocortin type 2 receptors of the adrenal gland, which stimulates the release of corticosteroids (CORTs) from its cortex (Klengel et al., 2014). These corticosteroids are commonly known as “stress hormones,” and are capable of binding to two intracellular receptors: mineralocorticoid receptors (MRs) and glucocorticoid receptors (GRs). Once bound to their hormone ligand, MRs and GRs act as transcription factors by migrating to the nucleus and binding to hormone response elements often found in gene promoters (Funder, 1997). Binding of corticosteroids to GRs in the pituitary and hippocampus inhibits the production of CRF, establishing a negative feedback loop. In the adrenal glands, this binding promotes the release of epinephrine and norepinephrine, two hormones involved in the fight-or-flight response (Edwards and Guilliams, 2010; Bartlett et al., 2017).

## Methodology

### Animal testing in neuroepigenetics

Elucidating molecular changes in the human brain is made difficult by the timing of sample acquisition, cell type distribution between samples, cause of death, and brain agonal state, as well as sample handling, storage temperatures, and sample size (Bakulski et al., 2016). Findings gleaned from postmortem brains must be considered carefully, as undocumented confounding factors such as recreational drug use and nonprescribed medicine abuse may bias results. Additionally, degradation of epigenetic markers, particularly histone acetylation, has been observed with more time elapsed postmortem (Jarmasz et al., 2019). Many epigenetic studies conducted in humans involve the retrieval of peripheral samples such as cord, systemic blood, or saliva (Schiele and Domschke, 2018). As such, much of neuroepigenetic psychiatric research relies on the use of animal models, particularly rodents.

Brain circuitry underlying anxiety, including neuronal pathways and neurotransmitters, is highly conserved between humans and rodents (Hohoff, 2009). Of course, replicating a psychiatric disorder in animals with underdeveloped cortices compared with that of humans, poses some difficulty when attempting to study anxiety holistically. Symptoms of mental illnesses are accompanied by emotional, cognitive, and motivational aspects that are not attributable to the lower mammal species being studied (Lezak et al., 2017). In 2009, the National Institute of Mental Health initiated the Research Domain Criteria project, with their stated goal as: “[to] transform the understanding and treatment of mental illnesses through basic and clinical research” (Cuthbert, 2014). As such, for different disorders, an accepted literature has emerged wherein several methods and tests have been accepted as standards for inducing/modeling and measuring anxiety-like or depressive-like symptoms and behaviors in animals, particularly in rodents. Of course, improper and inconsistent handling of rodents within a treatment group may skew any results obtained (Walf and Frye, 2007). One often runs the risk of overinterpreting data retrieved from animal studies when applying them to human psychiatric theory, since studying anxiety in animals requires a simplified, reductionist approach (Hohoff, 2009).

#### Modeling anxiety

To measure anxiety-like behaviors in animals, researchers must first create an anxiety model by applying different stressors to the animal. There are also a variety of rodents available that have been bred to have lower or higher responses to stress, or with different innate anxiety temperaments, with relevant anxiety outcomes, several of which are discussed in this review (Simmons et al., 2012; Chaudhury et al., 2014; Sotnikov et al., 2014). It is important to understand that while these stress paradigms very rarely mimic those experienced by humans, they evoke a relevant anxiety phenotype in the animal model. Stressors typically include restraint in a cylindrical, perforated tube, application of an electric shock to the foot (0.5–2 mA) for 1–2 s, maternal separation following birth, cage tilting, disrupted light/dark cycles, and food deprivation, to list a few (Campos et al., 2013). Several stress paradigms that permit anxiety modeling may be used, as follows in Table 1 (for review, see Lezak, 2017).

**Table 1:** Summary of stress models used to create anxiety models in rodents

Stress model	Description	Source
CMS; CUS; CVS	Originally developed by Paul Willner as a model for depression, involves the subjugation of animals to a series of multiple, unpredictable stressors over a prolonged period; May vary in the combination and duration of stressors; Many limitations, such as difficulty to replicate	Willner, 1997; Lezak et al., 2017
CSDS	Initially developed to model depression by Avgustinovich et al. (2005); was popularly adapted to investigate anxiety phenotypes by Krishnan et al. (2007); Study animal is introduced to the cage of a larger, aggressive animal (often a different strain of rat or mouse); Study animal is consider the “intruder”; aggressor animal is considered a “resident”; Exposure lasts for 10 min for 10 d, but may differ based on the researcher’s goals; Some animals may overcome CSDS-induced anxiety and are considered models for studying anxiety resilience; Limitations: sex-related differences (females tend not to participate in territorial related aggression); and injury to the model animal by the aggressor animal	Avgustinovich et al., 2005; Krishnan et al., 2007; Lezak et al., 2017
Prenatal stress	Originally used in rats by William Thompson, this stress model involves the application of a stressor to a pregnant dam; later adapted to study anxiety-like behaviors in offspring by other researchers (Vallée et al., 1997); Considered a developmental form of stress; Stressors include, e.g., footshock, restraint stress, subjection to EDCs; for more comprehensive reading on variations used in this model, please refer to Weinstock (2017)	Thompson et al., 1962; Vallée et al., 1997; Lezak et al., 2017
Postnatal stress	Administration of a stressor following the birth of pups and was first reportedly used by Krzysztof Janus (1987); Considered a developmental form of early life stress; The most commonly employed stressor is maternal separation in rodents, the time of separation is crucial for inducing anxiety-like phenotypes: separation at P3 to P4 tends to induce anxiety-like behaviors, while separation at P11 to P12 has been shown to cause the opposite effect of hyporesponsiveness	Murthy and Gould, 2018; Lezak et al., 2017; van Oers et al., 1998

CUS, Chronic unpredictable stress; CVS, chronic variable stress; P, postnatal day.

#### Measuring anxiety

Multiple tests are often used within a single study to observe anxiety-like behaviors. These tests measure either the avoidance behavior or defensive behavior of an animal, when a threat is perceived or introduced. Both of these behaviors imply anxiety-like phenotypes in these models. Furthermore, the use of rodent models in anxiety research has been consistently validated through the administration of drugs that exert anxiolytic and anxiogenic effects in humans, where they are shown to exert similar effects that are, in turn, measurable according to the tests discussed below (Hohoff, 2009). Note that there are a plethora of tests that can be used to measure anxiety, such as novelty-induced hyponeophagia, elevated zero mazes, Geller–Seifter and Vogel tests, and marble burying, not discussed here (Harro, 2018). The three most commonly used tests in the studies included in this review are summarized below in Table 2.

**Table 2:** Summary of assays used to measure the anxiety phenotype in rodent models

Test	Characteristics	Source
Light/dark box assay	Box apparatus is divided into two sections: the smaller dark “protected” side (minimally lit with black walls), and the larger light ‘unprotected’ side (brightly illuminated with white walls); Relies on the principle of the innate aversion of a rodent to light and exposure to predators as a prey animals; Shorter latency periods entering the light side, and/or longer periods spent here, are interpreted as reduced anxiety-like behaviors; Measures the approach–avoidance behavior	Crawley and Goodwin, 1980; Campos et al., 2013; Lezak et al., 2017
EPM test	Consists of two open, or unenclosed arms opposing two enclosed arms in the shape of a plus sign; Apparatus is elevated several feet from the ground; Animals are placed at the left of the EPM and allowed to explore freely for 5 min; Exposure created by open arms is associated with anxiety-like behavior, such as increased defecation and corticosteroid levels; More time spent in, as well as higher percentage of entries into the open arms of the EPM, are interpreted as reduced anxiety-like indices; Measures approach–avoidance behaviors	Handley and Mithani, 1984; Korte, 2001; Campos et al., 2013; Lezak et al., 2017
Open field test	Consists of an open box divided into layers of rings from the left of the box to the corners; The time spent in the middle where hypothetically the animal is most exposed and vulnerable, is compared with the amount of time spent hugging the safer corners of the box; The number of times ventured towards the left of the box is also recorded; Reduced anxiety is inferred if the animal tends to venture out from peripheral zones, or spend longer periods in the more central zones of the open box; Relies on instinctual fear responses to predators	Lezak et al., 2017

Avoidance behaviors measured in these three assays are homologous to the maladaptive avoidance behaviors observed in human anxiety disorders, where perceived threats, such as places and situations, are avoided (Hohoff, 2009).

Additionally, applications of pavlovian fear conditioning have led to the development of other assays that measure anxiety-like behaviors in animals. For example, pairing an aversive unconditioned stimuli, such as a footshock, with a tone/sound, light, or a context/environment (neutral conditioned stimuli) can elicit fear responses to the conditioned stimuli in the absence of the footshock on re-exposure to the conditioned stimulus (Lezak et al., 2017). Acoustic startle is a measurable “flinch” to a delivered white noise, that has an amplitude that can be quantified in units of force, and is hypothesized to reflect the state of alertness associated with increased anxiety (Lezak et al., 2017). Freezing behavior is the time an animal spends immovable/frozen in fear on application of the conditioned stimulus, and serves as a measure of anxiety-like behavior where behavioral inhibition is observed (Korte, 2001). Importantly, assays reliant on pavlovian fear conditioning are also applicable to post-traumatic stress disorder (PTSD) models, so results should be interpreted carefully (Hohoff, 2009).

#### Molecular tests

Within the field of epigenetics, several specialized assays have emerged transforming the way we conduct research in the 21st century. These techniques often require homogenized tissue samples, such as centrifuged blood, or rodent brain samples.

Bisulfite sequencing is used to identify methylated DNA (5mC) by converting all unmethylated cytosine bases to uracil through the addition of a bisulfate agent such as sodium bisulfate. This detection method does not work for identifying 5-hydroxymethlycytosine (5hmC). In a follow-up PCR, the uracil residues are converted to complement thymines, while the 5mC remains unconverted (Li and Tollefsbol, 2011). 5mC is then detectable by a subsequent RT-PCR step by use of methylation-specific primers (these vary per study), which will result in thousands of amplicons that can then be sequenced via next-generation sequencing or identified by whole-genome methylation arrays (Yong et al., 2016). Methylation content can also be assessed as a percentage at CpG sites, as a ratio of cytosine to thymine (Li and Tollefsbol, 2011). Darst et al. (2010) have described the five basic steps in bisulfite conversion of DNA, as follows: (1) denaturation of the DNA sample (∼2 μg genomic DNA); (2) addition and incubation with bisulfite agent at elevated temperatures (98°C) for deamination (conversion of cytosine to uracil); (3) desalting to remove the bisulfate; (4) desulfonation of sulfonyl uracil adducts in the sample DNA, which tend to form at alkaline pH; and (5) removal of the desulfonation solution used. Bisulfite sequencing provides a readout of the methylation status of every individual cytosine within a defined region of the genome, therefore permitting the identification of differentially methylated DNA between anxiety and control models as an example.

Chromatin immunoprecipitation (ChIP) is capable of detecting and mapping protein–DNA interactions, such as DNA-binding sites for specific proteins. These proteins include transcription factors and other chromatin modeling proteins such as DNMTs, but most importantly, they include histone modifications/markings (Park, 2009). ChIP may also be used to retrieve DNA with 5hmC marks (Papale et al., 2017). The first step of ChIP requires the temporary cross-linking of DNA with DNA-bound proteins using formaldehyde in the sample of interest. This ensures retrieval of the target DNA, and buffers against the loss of chromatin bound to the protein of interest. The chromatin is then sheared by sonication, where the sample is exposed to ultrasonic frequencies/vibrations. Here, the DNA fragments are ∼200–1000 bp long (Nelson et al., 2006). The subsequent success of ChIP relies on the validity and quality of specific antibodies chosen for the target protein being investigated, for example, anti-H3K27me. Antibodies are usually coupled to magnetic beads and immunoprecipitate with the histone mark being investigated by binding in a structure-specific manner—the overall principle of ChIP. The beads are retrieved using magnets, or centrifugation, depending on the bead type used, and the sample isolate, which now contains bead–antibody–protein–DNA target sequence complexes, is washed (Song et al., 2015). Any chromatin not bound to the target histone mark is ultimately lost when the target DNA is “pulled down” via the antibody-coated beads. The protein–DNA sequence is unlinked using proteinase K, which removes the antibodies and the target protein, and the sample is centrifuged to purify the DNA (Nelson et al., 2006). The retrieved DNA can then be amplified via PCR, and/or analyzed by hybridizing these fragments to a microarray (ChIP-chip). With technological advancements, however, pairing ChIP with next generation sequencing, commonly referred to as ChIP-seq, has proven to be far more beneficial for the purpose of epigenetic studies. Though ChIP-seq is more costly, it requires less DNA input (Park, 2009). Sequencing of the target DNA allows researchers to determine what genes are under the control of specific histone marks. Pairing ChIP-seq with Western blots and immunofluorescent or immunohistochemical assays, which detect upregulation or downregulation of specific histone marks in a sample tissue, allows researchers to formulate relationships between a differential histone mark and an observed change in gene expression.

## Findings

Target genes and protein products of these neuroepigenetic studies in anxiety models are numerous and variable. Table 3 below summarizes these targets and their abbreviations for more comprehensive reading.

**Table 3:** Summary of gene abbreviations

Protein product	Gene denotation
Glutamate receptor subunits	*Grik1*, *Grik2*, *Grin1*, *Grin2b*, *Grin3a*, *Grm5*, *Nr2b*
GABA receptor subunit	*Gabra2*, *Gabbr1*, *Gabbr2*
Mineralocorticoid receptor	*Nr3c2*
Glucocorticoid receptor	*Nr3c1*
Jagged-1	*JAG1*
B-cell lymphoma/leukemia 11A	*Bcl11A*
Corticotropin-releasing hormone/corticotropin-releasing factor	*CRH*/*CRF*
Glutamic acid decarboxylase	*GAD67*
CRH receptor	*Crhr1*
FK506 binding protein 5	*FKBP5*
Histone-lysine *N*-methyltransferase SUV39H1	*SUV39H1*
Euchromatic histone-lysine *N*-methyltransferase	*EHMT2*, *G9a*
G9a-like protein	*EHMT1*, *GLP*

### DNA methylation

DNA methylation plays crucial roles in gene-silencing events, particularly at the promoter regions of genes, and varies per tissue type (Ohgane et al., 2008). Caution should be taken when comparing patterns of methylation as well as the resulting gene expression differences in peripheral samples to CNS samples of different brain regions, and when comparing different brain regions. Within the scope of DNA methylation studies, studies may focus on the presence of DNMTs, without elaborating on specific genes affected by any abnormal levels of these enzymes measured, while other studies may focus on promoter-methylated trends of genes implicated in AD pathophysiology, where the DNMTs responsible for differential gene expression patterns were not necessarily investigated.

#### DNMT expression

Investigations into the patterns of DNA methyltransferase expression offer insight into to how these enzymes are able to respond to external stimuli to epigenetically modify gene expression in the CNS. Expression levels of DNMTs positively correlate to DNMT activity and thus to any global methylation trends observed (Slack et al., 1999). While DNMT expression levels are important to investigate, failure to study the connection between their expression trends and methylation trends of anxiety-related genes in different brain regions, makes pathophysiological conclusions difficult to formulate, though it leaves room for future research directions.

Recent studies have reported anxiety-like behaviors in adult mice following prenatal deletion of *Dnmt1* from neural stem cells. Further analysis to uncover the downstream effects of differential *Dnmt1* expression has not yet been conducted with regard to anxiety (Noguchi et al., 2016). In a previous study, Low Novelty-Responding (bLR) rats, bred to exhibit increased anxiety and depressive-like behaviors, displayed decreased mRNA levels of Dnmt1 in the dentate gyrus (DG) and CA3 of the hippocampus, compared with their High Novelty-Responding (bHR) counterparts (Simmons et al., 2012). This DG–CA3 circuitry is believed to be responsible for event sequence-related memory formation and fear learning, which underlies the anxiety phenotype (McEwen et al., 2012; Zhang et al., 2014). Overall, these studies indicate that decreased levels of Dnmt1 underlie the anxiety phenotype. It can be postulated that this may be a result of reduced global methylation.

Previously, significantly reduced expression of *DNMT3a* was detected in blood samples retrieved from an anxious cohort consisting of young adults that correlated directly with anxiety severity (Murphy et al., 2015). Individuals of the anxious cohort also displayed higher levels of global methylation compared with nonanxious individuals, though site-specific methylation trends were not assessed (Murphy et al., 2015). In another study, C57BL/6J mice exposed to aggressive CD1 mice to induce chronic social defeat stress (CSDS), displayed selective downregulation of *Dnmt3a* in their medial prefrontal cortex (mPFC; Elliott et al., 2016). This correlated with a significant reduction in global DNA methylation of the mPFC, contrary to the increased methylation observed in human blood samples. While an increase in blood methylation may serve as a biomarker for anxiety, this methylation increase may not necessarily be observed in the brain.

More specifically, Dnmt3a1, a splice variant of Dnmt3a, was significantly reduced in CSDS mice, with no relevant changes in Dnmt3a2 (Elliott et al., 2016). Most interestingly, the researchers found a negative glucocorticoid response element sequence upstream of *Dnmt3a1* TSS, which specifically binds to *NR3C1*, the gene that encodes for the GR, which binds to the stress hormone cortisol to regulate HPA axis response, suggesting a possible pathway through which Dnmt3a may exert anxiolytic effects when expressed. (Elliott et al., 2016). Viral knockdown of *Dnmt3a* induced the same anxiety-like phenotype previously observed in CSDS mice as measured by the elevated plus maze (EPM) test, while *Dnmt3a1* viral overexpression in mouse dorsal mPFC, which regulates fear, anxiety, risk taking, and decision-making, rescued CSDS-induced anxiety (Chocyk et al., 2013; Elliott et al., 2016). This suggests that Dnmt3a in the mPFC plays a pivotal role in the development of anxiety. Specific genes that are regulated by Dnmt3a in the mPFC, and thus, how enzyme knockdown induces the anxiety phenotype, remain elusive. Ontological and functional analysis of genes expressed at sites of differential Dnmt3a activity would help to further elucidate its specific role in the development of the anxiety phenotype.

A novel study comparing juvenile mice on low-methyl diets versus normal diets, investigated the effects of methyl deficiency on DNA methylation and Dnmt expression. Interestingly, researchers found that mice lacking methyl donors displayed decreased expression levels of both Dnmt3a and Dnmt3b in the hippocampus, a finding that correlated with impairment in hippocampal fear memory acquisition and reduced anxiety-like behaviors, as well as a decrease in the expression of *Grin2b*, a glutamate receptor involved in excitatory pathways (Ishii et al., 2014; Nuss, 2015). Of note, there was a moderate increase in *Grin1* expression, later observed in rhesus monkeys discussed in the Other genes subsection below. These expression levels were reversed on the administration of a normal diet, though anxiety-like behaviors became elevated. This particular study highlights the role of DNMTs in fear memory consolidation and plasticity in the hippocampus at younger ages, possibly forming the core psychopathology of inappropriate anxiety responses that may carry into adulthood (Ishii et al., 2014).

Last, knockdown of *Dnmt3a* in the mPFC of rats resulted in an anxiety-like phenotype in the study by Elliott et al. (2016), contrary to the reduced anxiety behaviors observed following a decrease in *Dnmt3a* expression in the mouse hippocampus in the research of Ishii et al. (2014). This suggests that even when DNMT expression patterns are similar, the consequences of these trends vary per brain region when influencing the anxiety phenotype. We can hypothesize that the collection of genes regulated by Dnmt3a in the hippocampus, differ from those of the mPFC.

#### NR3C1 and FKBP5

The *NR3C1* gene is composed of multiple exons and codes for the GR, which binds the hormone cortisol in a pathway that regulates the HPA axis response during stress. Of interest to researchers is the methylation status of this gene as well as its overall expression patterns that underscore the anxiety phenotype. Previous studies have shown that prenatal exposure to maternal depression and increased cortisol levels significantly increase methylation of the *NR3C1* gene in neonatal cord blood samples at exon 1F (Oberlander, 2008; Hompes et al., 2013). Data from the TRAILS (Tracking Adolescents’ Individual Lives Survey) study in Dutch adolescents (mean age, 16 years) showed similar hypermethylation at exon 1F in whole-blood samples of individuals who reported stressful life events (SLEs) in childhood and adolescence, including sexual abuse and other trauma (van der Knaap et al., 2014).

However, divergent research has shown hypomethylation of exon 1F in the promoter region of *NR3C1* in leukocyte blood samples from individuals (age range, 18–65 years) who experienced adverse childhood events. Additionally, individuals diagnosed with an AD who did not report adverse childhood events, showed a similar trend of reduced methylation (Tyrka et al., 2016). This suggests that early stressors in childhood may epigenetically poise an individual toward anxiety pathology, where a decrease in methylation correlates to an increase in GR expression and overall hyperactivity of the HPA axis (Tyrka et al., 2016). These human studies leave much to be desired, insofar as they do not address the anxiety phenotype, or measure GR expression levels.

In consensus with increased methylation studies of *NR3C1*, hypermethylation of exon 1F at several CpG sites correlated with a decrease in the mRNA levels of GRα (one isoform of glucocorticoid receptor) in samples of peripheral blood mononuclear cells (PBMCs) of adults diagnosed with generalized AD (GAD; Wang et al., 2017). Higher levels of serum cortisol were also detected in GAD individuals. Of note, >50% of the GAD group had comorbid depression and ∼42% smoked (Wang et al., 2017). Wang et al. (2017) argue that Tyrka et al. (2016) failed to homogenize the population sample (e.g., by AD diagnosis) and that the use of childhood traumatic experiences (CTEs) as a criterion adds uncontrollable variability to the results, though arguably, the Wang et al. (2017) study is also confounded by comorbid depression and substance use. Individuals in the Wang et al. (2017) study who reported CTEs demonstrated lower levels of methylation compared with non-CTE GAD individuals. These researchers argue that reduced GR expression because of *NR3C1* hypomethylation, promoted HPA axis hyperactivity, and increased cortisol production as a result of decreased negative feedback regulated by GRs (Wang et al., 2017).

In concordance with the hypomethylation hypothesis, however, N3RC1 heterozygote mice (*NR3C1*^+/−^) with depleted levels of GRs showed a significant increase in anxiety-like behaviors, but not depressive-like behaviors. Additionally, hypomethylation of *FKBP5*, which encodes FK506 binding protein 5, a proximal protein regulator of GRs that has been shown to decrease GR affinity for its ligand cortisol, therefore disrupting the negative feedback loop in the HPA axis, was reported in the placenta (Wochnik et al., 2005; Schmidt et al., 2019). Overexpression of *FKBP5* as a result of decreased *FKBP5* methylation in the amygdala is associated with the anxiety phenotype in adult rats (St-Cyr et al., 2017). Additionally, FKBP51 knock-out mice are also more resilient to CSDS (Hartmann et al., 2012). In a follow-up study, researchers virally overexpressed mutant FKBP51 in the BLA, which is involved in pavlovian fear learning and receives sensory input from the parietal, cingulate, and prefrontal cortices, elicited anxiety-like behaviors in mice (Pessoa, 2010; Hartmann et al., 2015). Treatment with Ligand2, an antagonist specific to mutant FKBP51, had significant anxiolytic results in these mice, measured by the open field, EPM, and light/dark box tests. Another inhibitor, SAFit2, which is capable of inhibiting wild-type FKBP51, also reduced anxiety-like behaviors 16 h following administration in naive adult mice following either peripheral or BLA-injected administration (Hartmann et al., 2015). These findings suggest that FKBP51 inhibitors may be used as a potential pharmaceutical intervention for anxiety across demographics.

In humans, decreased levels of *FKBP5* methylation detected in blood samples was found to be associated with better cognitive behavioral therapy (CBT) treatment outcomes from pretreatment to post-treatment patients formally diagnosed with phobias. Meanwhile patients with no changes or increased levels of *FKBP5* methylation had poorer therapy outcomes in comparison (Roberts et al., 2019). Similar findings, with decreased *FKBP5* methylation detected in saliva samples associated with better CBT outcomes, were also reported in a cohort of children diagnosed with anxiety between the ages of 8–15 years (Roberts et al., 2015). While these findings contradict data retrieved from the rodent models in studies by St-Cyr et al. (2017) and Hartmann et al. (2015) where decreased methylation and subsequent increase in FKBP5 expression underscored the anxiety phenotype, these studies suggest that *FKBP5* methylation levels in the blood and saliva can be used to determine populations that may benefit from aggressive CBT regimens.

#### GABA

GABA, the main inhibitory neurotransmitter of the CNS, is another much studied candidate gene in anxiety research. In newborns of pregnant mothers that experienced anxiety measured by PRAQ, a pregnancy-related anxiety questionnaire, researchers found that an increase in methylation of CpG islands of *GABBR1* in the cord blood of male newborns (GABA_B_ receptor subunit 1 gene), was associated with higher anxiety levels of pregnant mothers as well as increased cortisol levels in these infants on vaccination (applied stressor; Vangeel et al., 2017). Similar methylation trends were observed at the *NR3C1* gene in the previously discussed neonatal cord blood study further validating the impact of prenatal stressors *in utero* on the methylation status of genes in newborns (Oberlander, 2008; Hompes et al., 2013).

Studies of methylation trends pertaining to GABA-associated genes in animals permit us to further study the consequences of aberrant expression levels. In one study, researchers used H67D male mutant mice that contained increased levels of redox-active iron in the brain. They found that with an increase in brain iron load, global methylation, Dnmt1 mRNA levels, and activity, were all decreased (Ye et al., 2018). Additionally H67D mutant mice with decreased *Dnmt1* expression exhibited lower levels of anxiety in the EPM assay compared with wild-type counterparts. However, these findings contradict those of the study by Simmons et al. (2012) in anxious bLR rats as well as Noguchi’s prenatal *Dnmt1* deletion mice, where reduced *Dnmt1* expression led to an increase in the anxiety phenotype. The investigators found an increase in *Gabra2* (GABA_A_ receptor subunit 2) mRNA levels by 140% in the mutant mice, as well as an overall decrease in GABA with a decrease in global methylation and *Dnmt1* expression and activity. Whether the increase in *Gabra2* expression is because of decreased methylation and reduced *Dnmt1* expression, or to the reduction in GABA, remains unclear (Ye et al., 2018). Binding sites for Dnmt1 on the *Gabra2* gene can be pursued in future studies.

Contradictorily, a study conducted in mice fed a methyl-deficient diet, showed the opposite effects, such that a decrease in the expression of *Gabra2* in mouse hippocampus correlated with a decrease in anxiety-like behaviors, although hippocampal and whole-brain studies are difficult to compare. Furthermore, though *Dnmt3a* and *Dnmt3b* showed significant reduction in expression, these levels do not explain a decrease in Gabra2 levels, and DNMT1 levels were unaffected in low methyl-fed mice (Ishii et al., 2014). This suggests that *Gabra2* is under the regulation of multiple epigenetic factors and not a single independently acting enzyme like a DNMT.

While the Ye et al. (2018) iron study failed to investigate levels of *Dnmt3a* and *Dnmt3b*, the study by Ishii et al. (2014) failed to correlate decreases in *Gabra2* levels with detected GABA levels. As previously mentioned, harmful prenatal exposures include endocrine disrupting chemicals (EDCs) such as bisphenol-A (BPA). In one study using rat dams fed BPA, newborns showed an increase in Dnmt1 mRNA expression levels in their BLA in tandem with increased anxiety behavior observations (Zhou et al., 2013). Researchers believed that the GABAergic pathway was affected, because an increase in Dnmt1 correlated with a decrease in glutamine decarboxylase (GAD67) mRNA, the enzyme responsible for the production of GABA from glutamate, though GABA and glutamate levels were not directly measured (Valenzuela et al., 2011; Zhou et al., 2013). This anxiogenic effect of increased Dnmt1 in the BLA parallels the anxiolytic effect of decreased Dnmt1 in the H67D mice of the study by Ye et al. (2018). Researchers also showed that the reversal of decreased GAD67 mRNA expression and subsequent anxiety-like behaviors in BPA rats is possible by administering 5-ada-Cdr, a hypomethylating agent (Zhou et al., 2013). The rescuing effect of 5-ada-Cdr was then later inhibited by use of picrotoxin, an antagonist of the GABA_A_ receptors (Zhou et al., 2013). Together, these findings show a clear role for decreased GABA in the creation of the anxiety phenotype, and that the multiple epigenetic changes underscoring this decrease provide potential therapeutic targets when considering treatments.

In a follow-up study published 5 years later, the activity of glutamatergic pathways in relation to GABAergic inhibition, is somewhat elucidated in mice. Using a prenatal restraint stress (PRS) model on pregnant dams, researchers found that PRS offspring displayed similar anxiety-like behaviors as BPA-treated mice, as well as an increased binding of overexpressed Dnmt1 to the promoter region of *Gad67* (along with MeCP2, discussed later). This provided a direct relationship between the decreased mRNA levels of GAD67 (repressed by promoter hypermethylation) and overexpressed Dnmt1 (increased methylating activity), as well as underscoring a direct role of prenatal stressors *in utero* and later observed anxiety phenotypes in offspring (Zhu et al., 2018). Most interestingly, these researchers conducted electrophysiological analysis on brain slices of PRS and control mice and found that on stimulation of the entorhinal cortex, PRS mice displayed a greater number of population spikes in the BLA. These findings were attributed to higher neuronal firing rates and cortical–BLA synaptic activity, suggesting that a decrease in GAD67 expression impairs GABAergic pathways, which in turn fail to inhibit glutamatergic or excitatory pathways in the BLA (Zhu et al., 2018). Manipulation of GAD67 expression by administering drugs that target the epigenetic markers regulating its expression, provides an exciting avenue for future treatment possibilities.

#### CRH/CRF-related genes

The *CRH*/*CRF* gene codes for corticotropin-releasing hormone/corticotropin-releasing factor and therefore plays a pivotal role in the regulation of the HPA axis and stress responses involved in anxiety. Prolonged demethylation of the *Crf* promoter in adult mice that displayed social avoidance has been reported in a CSDS model, accompanied by a subsequent increase in *Crf* mRNA levels in the PVN of the hypothalamus of these animals. Both findings were significantly reversed on administration of the antidepressant imipramine (Elliott et al., 2010). Researchers also detected decreased levels of *Dnmt3b*, but, more interestingly, it was observed that the viral knockdown of *Crf* in the PVN buffered against social avoidance behavior in CSDS mice. This suggests that increased *Crf* expression may underscore increased social anxiety behaviors in these animals by inducing HPA axis hyperactivity (Elliott et al., 2010).

Prenatal stress has also been shown to alter methylation states of *CRH* as previously shown in *GABA* and *NR3C1* cord blood sample studies. New mothers exposed to war conditions in the Democratic Republic of Congo exhibited different CRH methylation patterns based on the type of stress reported: war stress or chronic stress, highlighting that the stress type experienced influences the epigenetic change observed (Kertes et al., 2016).

Male rats born to dams subjected to PRS showed an increase in anxiety-like behaviors on assessment with open-field and EPM tests, as well as higher serum levels of corticosterone. PRS offspring also showed higher corticosterone concentrations when subjected to their own restraint stress session compared with the control group, indicating HPA axis hyperactivity in PRS offspring (Xu et al., 2014). In the hypothalamus, CRH mRNA expression was decreased in the PRS group before restraint stress administration, but increased significantly following the restraint stress. This suggests that *in utero* exposure to elevated maternal corticosterone concentrations epigenetically primed PRS offspring for later *Crh* overexpression when exposed to stress (Xu et al., 2014). These findings suggest that before birth, an individual may already be more at risk of developing an AD based on the physical and mental state of the mother during the pregnancy. PRS offspring also exhibited decreased *Crh* promoter methylation in the hypothalamus compared with control animals, suggesting that the recorded increase in corticosterone is a result of HPA axis hyperactivity and failure to decrease *Crh* expression through the negative feedback loop (Xu et al., 2014).

Another prenatal stressor, gestational hypoxia (GIH), induced anxiety-like behaviors in newborn male rats. In the hypothalamic PVN, an increase in CRH and CRHR1 (a CRH receptor gene) was observed in male offspring, but not females, suggesting a sex-related positive stress adaptation in female animals (Wang et al., 2013). In both 19-d-old male embryos and 90-d-old male GIH offspring, hypomethylation of CpG islands within the *Crhr1* promoter were observed, suggesting that the hypomethylation of *Crhr1* initiated *in utero* persists even after birth into adulthood (Wang et al., 2013). Similar findings were observed in peripheral blood samples of patients diagnosed specifically with panic disorder, making *Crhr1* hypomethylation a possible diagnostic marker for panic disorder and other ADs (Schartner et al., 2017). To better understand this trend of hypomethylation, Wang et al. (2013) reported that while *Dnmt1* and *Dnmt3a* were unaltered in male and female GIH embryos, contrary to aforementioned anxiety studies (though different stress paradigms and brain regions were used), DNMT3B was downregulated in male embryos and upregulated in female embryos, possibly explaining the methylation differences of *Crhr1* in male and female offspring. The decreased expression of DNMT3B in the PVN persisted into adulthood in 90-d-old male GIH rats (Wang et al., 2013). The CRH/Crf studies of both Wang et al. (2013) and Elliott et al. (2010) show a direct correlation between Dnmt3b levels and CRF-associated genes in the PVN, suggesting that in this brain region, Dnmt3b may be responsible for modulating their expression via methylation. The effect of *Dnmt3b* knockout in PVN cells on the methylation state of CpG sites in the *Crhr1* promoter would be an interesting future study. We can hypothesize that a decrease in methylation of the *Crhr1* promoter leads to overexpression of *Crhr1* and overall HPA axis hyperactivity. ChIP-seq can be used to elucidate binding sites of Dnmt3b to CpG sites in the promoters of *Crf* and CRF-related genes since there appears to be a strong link between decreased *Dnmt3b* and *Crf* expression. In another study using high-anxiety behavior (HAB) and low-anxiety behavior (LAB) mice, bred for innate levels of anxious demeanors (not to be confused with bHR/bLR animals), researchers reported that not only do HAB mice display an overexpression of CRHR1 (CRH receptor) in the basal amygdala compared with LAB mice, but that exposure of HAB mice to an enriched environment (EE) positively stimulates the animal, reversing this expression. Additionally, the exposure of LAB mice to chronic mild stress (CMS) shifted CRHR1 expression levels to that of anxious HAB mice (Sotnikov et al., 2014). These preliminary findings demonstrate a direct impact of the external environment on the anxiety phenotype by modulating gene expression. The use of a CRHR antagonist in HAB mice had an anxiolytic effect, further supporting the role of overexpressed Crhr in various brain regions in observable anxiety phenotypes (Sotnikov et al., 2014). Interestingly, EE in HAB mice and CMS in LAB mice both caused an increase in methylation at CpG1 upstream of the *Crhr1* promoter in the amygdala. However, recall that in the study of Wang et al. (2013), hypomethylation of this gene region in the hypothalamus (not amygdala) promoted an anxiety-like phenotype. In the study by Sotnikov et al. (2014), hypermethylation of CpG1 in the *Crhr* promoter can increase, or decrease, anxiety-like behaviors, emphasizing that gene expression regulation is incredibly complex as other epigenetic modifications may cooperate to regulate gene expression, which we will see below.

In an attempt to explain how a unidirectional epigenetic alteration can underscore a bidirectional shift in anxiety phenotypes, the researchers honed in on a transcription factor Ying-Yang1 (YY1), which was reported to be bidirectionally expressed between HAB-EE and LAB-CMS mice (Sotnikov et al., 2014). YY1 was found to bind near CpG1 of the promoter of *Crhr1* in response to the methylation of CpG1. In HAB-EE mice, lower levels of YY1 expression coupled with an increase in methylation correlated with a downregulation of Crhr1 expression. An increase in YY1 in LAB-CMS with an increase in methylation, correlated with the upregulation of Crhr1 expression (Sotnikov et al., 2014). Expression of YY1 may be attributed to EE (downregulation) and CMS (upregulation) as positive and negative stressors, respectively. Overexpression of YY1 in mouse neuroblastoma cells (N2a) was shown to enhance CRHR expression by increased promoter activity, suggesting that increased methylation at CpG1 of *Crhr1* in LAB-CMS anxiety-exhibiting mice does not repress CRHR expression when YY1 expression is increased (Sotnikov et al., 2014). Identification of this pattern of differential methylation of the *Crhr1* gene highlights brain plasticity, such that external stimuli, such as EE and CMS, are able to alter gene methylation states and subsequently induce changes in anxiety-like behaviors via chromatin remodeling protein recruitment (Sotnikov et al., 2014). Whether YY1 recruits histone-modifying complexes to the *Crhr1* gene in these models would be an interesting follow-up study, as well as elucidation of any protein–protein interactions between YY1 and DNMT3B.

To summarize, though there is consensus that the overexpression of CRH/CRF-related genes in the amygdala and hypothalamus underscores HPA axis hyperactivity, and therefore contributes to the observed anxiety phenotype, elucidation of the epigenetic regulation of these genes is crucial toward pinning down anxiety-specific targets for therapy. Findings such as differential methylation trends and the expression levels of YY1 and Dmnt3b, highlight the need to pool existing literature to encourage more cohesive and expansive studies within the field.

#### BDNF

BDNF contains several functional exons, and its expression levels, transcripts, and associated epigenetic markers vary per brain region by use of alternative splicing and promoters (Cattaneo et al., 2016). This makes it exceedingly difficult to characterize into simple, straightforward trends when studying psychopathologies. Recall that BDNF is a neurotrophic factor that facilitates neurogenesis globally in the CNS (Cattaneo et al., 2016). We can therefore surmise that BDNF expression is beneficial based on the brain region and the timing of plasticity changes because of this neurogenesis, such as during periods of fear learning versus positive safety appraisal events. Several studies agree that BDNF serum levels fail to act as biomarkers for anxiety since dysregulated expression of this factor is present in multiple psychiatric illnesses including depression and schizophrenia (Molendijk et al., 2012; Carlino et al., 2015; Cattaneo et al., 2016). However, understanding the impact of DNA methylation states of *BDNF* is still crucial for understanding *BDNF* expression modulating the underlying neurophysiology of psychiatric illnesses. Like CRH, GABA, and NR3C1, prenatal factors such as maternal depression may impact BDNF levels in the cord blood of neonates such that BDNF concentrations were significantly lower compared with healthy control subjects (Sonmez et al., 2019). The actual impact of decreased BDNF in these newborns can only be unearthed by follow-up studies that track these children over their life span. Given the role of BDNF in brain plasticity, memory, and learning, we can conclude that levels of BDNF at different developmental stages may influence resilience and vulnerability to anxiety during those developmental stages. A recent review by Poon et al. (2021) summarizes the potential in targeting BDNF expression with antidepressants to facilitate fear memory extinction in a depression paradigm that may also be used to alleviate anxiety-related pathology.

#### Other genes

Though rarely conducted, a single anxiety study on 23 rhesus macaque monkeys (age, ∼1.3 years) matched phenotypically for anxious temperament (AT), analyzed genome-wide DNA methylation and mRNA expression in the CeA (Alisch et al., 2014). Primates such as these are the best model for investigating relevant anxiety traits in humans, since they share genetic, neuronal, and phenotypic foundations of complex socioemotional behaviors demonstrated by humans. DNA methylation analysis of these AT monkeys revealed genome-wide hypomethylation of CpG islands in promoter regions, particularly in TSSs (<10%), with higher methylation levels further away from the TSSs (Alisch et al., 2014). Researchers also identified almost 5500 CpG sites with AT-associated methylation, 87% of which showed a decrease in methylation levels with an increase in AT severity. Further analysis revealed that AT-associated methylation events were more prominent in the gene body (55%) with very little representation at the gene promoter (0–2%; Alisch et al., 2014). Gene ontological and functional analysis of AT-associated loci and changes in gene expression, included most notably *GRIN1* and *GRM5*, which code for specific subunits of different glutamate receptors. The genes *JAG1* and *BCL11A* showed methylation patterns that predicted gene expression in the AT phenotype. *BCL11A* codes for a protein involved downstream of a glutamate receptor cascade for dendritic arborization, such that a decrease in its expression resulted in extreme AT (Alisch et al., 2014). *JAG1* on the other hand, acts as a ligand in NOTCH signaling, which plays a critical role in CNS development, including synaptic plasticity and memory formation. An increase in methylation was associated with a decrease in *JAG1* expression and an increase in AT severity (Alisch et al., 2014). Comparatively, differential DNA methylation of *JAG2* was reported in blood samples of twins (Alisch et al., 2017). *DMNT* expression levels were not investigated in the macaque study, so the enzymes and mechanisms responsible for the reported differential DNA methylation regions in the CeA were not defined.

Overall, there are a plethora of genes involved in the creation and persistence of the anxiety phenotype. For example, serotonin-associated genes, such as receptor genes, including *5-HTT*, have been thoroughly reviewed within the scope of mood disorders by Lesch (2011) in “When the serotonin transporter gene meets adversity: the contribution of animal models to understanding epigenetic mechanisms in affective disorders and resilience.”

Though a few major genes were discussed above in detail, as these are more popularly investigated, it is important to remember that no single isolated gene or individual epigenetic modification causes AD. Genome-wide studies, such as those of Alisch et al. (2014, 2017), not only provide a great overview of the complex gene patterns that underscore anxiety, but also generate a list of potential novel gene targets, especially those associated with excitatory and inhibitory CNS signaling pathways, for future studies.

The DNA methylation patterns discussed in the above sections are summarized in Table 4 below.

**Table 4:** Summary of DNA methylation patterns and differential gene expression levels

Marker	Sample retrieved	Anxiety model	Reference
↕*Dnmt1*	Hippocampus (CA1, CA3) Amygdala (medial, basolateral and lateral nucleus)	bLR animals (rats: P7 to P14)	Simmons et al., 2012
↓*Dnmt3a*	Peripheral blood	Anxious young adults (measured with HADS-A)	Murphy et al., 2015
	mPFC	Adult CSDS (9 weeks) mice (killed 4 weeks after CSDS)	Elliott et al., 2016
↓*Dnmt3a* ↓*Dnmt3b* ↓*Grin2b* ↓*Gabar2* ↑*Grin1*	Hippocampus	Low-methyl diet adult mice (6 and 12 weeks) (low anxiety)	Ishii et al., 2014
↑*NR3C1*1F (Me)	Peripheral whole blood	Adolescents (mean age = 16 years) who reported SLE's	van der Knaap et al., 2014
↓*NR3C1*1F (Me)	Leukocytic blood	Adults (age range = 18–65 years) who reported SLEs and diagnosed with variable ADs	Tyrka et al., 2016
↑*NR3C1* (Me) ↓GRα	PBMCs	Adults (mean age = 35 years) diagnosed with GAD	Wang et al., 2017
↓GR ↑*FKBP5*	Placenta	NR3C1^+/−^ mice	Schmidt et al., 2019
↑*FKBP5*	Amygdala	Adult rats (25 weeks) born to dams exposed to predator odors (prenatal/*in utero* stress)	St-Cyr et al., 2017
	Amygdala (basolateral)	Viral overexpression of FKBP51	Hartmann et al., 2015
↓*Dnmt1* ↑*Gabra2* ↓*GABA*	Whole brain	H67D male mutant mice (low anxiety)	Ye et al., 2018
↑*Dnmt1* ↓*GAD67*	Amygdala (basolateral)	Female mice (P45) exposed to BPA *in utero*	Zhou et al., 2013
		Female mice (P60/P70) exposed prenatally/in utero to maternal restraint stress	Zhu et al., 2018
↑*Crf* ↓*Dnmt3b*	Hypothalamus (PVN)	Adult CSDS mice	Elliott et al., 2010
↑*Crf* ↑Cortisol	Hypothalamus	PRS adolescent rats (P38) born to dams subjected to restraint stress	Xu et al., 2014
↑*Crf* ↑*CRHR1,* ↓*Dnmt3b*	Hypothalamus (PVN)	Male adult (P90) rats exposed *in utero* to gestational hypoxia	Wang et al., 2013
↓CRHR1 (Me)	Peripheral whole blood	Adults (mean age = 35 years) diagnosed with panic disorder	Schartner et al., 2017
↑*Crhr1*	Amygdala (basolateral)	HAB mice bred for a high anxiety trait, LAB mice exposed to CMS	Sotnikov et al., 2014
*GRIN1, GRM5* ↓*BCL11A,* ↓*JAG1*	Amygdala (central)	Anxious temperament rhesus macaques (mean age = 1.3 years)	Alisch et al., 2014

Genes are italicized. (me) denotes methylation reported. HADS-A, Hospital anxiety and depression scale; P, postnatal day.

### MeCP2: bridging DNA methylation and histone modifications

MeCP2 is known to act as a bridge between DNA methylation marks and HDACs, enzymes involved in histone modifications and has been shown to dock at CpG-methylated sites and recruit HDACs to the chromatin, forming a silencing complex that represses gene transcription (Martinowich et al., 2003). This binding is partially impaired if CpG sites are hypomethylated (Martinowich et al., 2003). Following depolarization of mouse embryonic day 15 (E14) cortical cells, MeCP2 was found to be partially dissociated from the *Bdnf* promoter, suggesting that neuronal firing may redistribute MeCP2 binding allowing for BDNF expression (Martinowich et al., 2003). In a study published a few years later, viral deletion of MeCP2 in the BLA of mice resulted in an increase in anxiety-like behaviors. This was accompanied by an increase in H3 acetylation, indicating that MeCP2 may be required to recruit HDACs to maintain lower levels of acetylation necessary for gene repression (Adachi et al., 2009). This finding was further supported on inhibiting HDACs with Trichostatin A (TSA), which resulted in reduced postsynaptic excitatory firing of cortical pyramidal neurons, a finding that paralleled MeCP2-null cells in the study by Adachi et al. (2009). Overall, it appears that MeCP2 is able to modulate synaptic transmission frequency through transcriptional repression via HDAC recruitment (Kavalali et al., 2011).

### Histone modifications

Histone modifications within the CNS play key roles in both memory formation and consolidation as seen in the animal studies discussed below (Day and Sweatt, 2011). Most anxiety-based studies hone in on histone acetylation and methylation marks pertaining to differential gene expression, though other histone markers exist, such as phosphorylation, ubiquitylation, serotonylation, and dopaminylation, of which the latter two are fairly new in the field of epigenetics, and have yet to be explored in any anxiety study (Berger, 2002; Farrelly et al., 2019). HDACs, the enzymes responsible for histone deacetylation, are usually associated with transcriptionally silent chromatin and are often the easiest to study as numerous HDAC inhibitors are available for use in anxiety animal models (de Ruijter et al., 2003). As we discuss differential histone modifications below, it is important to remember that any observed changes can impact multiple transcripts at once.

#### Histone marks and gene regulation

In a study using footshock paired with white noise in C57BL/6 mice (a model for PTSD and anxiety), animals that displayed fear extinction where the induced fear behavior such as freezing is lost by extinction training had increased levels of histone H4 acetylation around the promoter of *Bdnf* exon IV, compared with both naive and fear-conditioned animals without extinction controls. This hyperacetylation was concomitant with an increase in BDNF exon IV mRNA in the PFC of mice that achieved fear extinction (Bredy et al., 2007). It is likely that an increase in BDNF expression facilitated by H4 hyperacetylation underscores improved learning and subsequent extinction of fear-conditioned behaviors. Valproic acid (VPA), an HDAC inhibitor and mood stabilizer, was shown to potentiate long-term memory for fear extinction, suggesting that HDACs may perpetuate repressed *Bdnf* expression in anxiety models (Bredy et al., 2007).

In another BDNF-focused study, researchers reported a decrease in repressive H3K9me2 at the promoter of exon IV in the hippocampus of male rats exposed to maternal separation [early stress (ES)] following birth, which persisted for 2 months. This was accompanied by an observable increase in neurogenesis in the hippocampal DG and improved performance on the Morris Water Maze stress-associated test, indicating better spatial learning propensities (Suri et al., 2013). The opposite is observed in adulthood at 15 months, where increased H3K9me2 at the *Bdnf* IV promoter, decreased hippocampal expression of *Bdnf IV* and subsequent neurogenesis were observed in ES animals (Suri et al., 2013). These findings suggest that there is an inverse relationship with H3K9me2 and BDNF. The researchers believed that increased *Bdnf* expression in early life may facilitate fear learning and avoidance behaviors, while reduced expression and brain plasticity in later life may impair fear extinction (Suri et al., 2013). The biphasic changes in hippocampal plasticity, memory, and learning impairments observed in middle-aged animals appears to support the conclusion that while some epigenetic alterations may prove to be potentially adaptive in early developmental ages, shifts in histone methylation may have deleterious outcomes later in life (Suri et al., 2013). The antidepressant amitriptyline was able to attenuate H3K9me2 increase and subsequent *Bdnf* IV expression decrease, as well as accompanying cognitive decline, suggesting that these drugs may bolster cognitive behavioral therapy geared toward fear extinction learning (Suri et al., 2013). Other stress models have shown similar histone methylation trends in the hippocampal DG, such as in rats subjected to chronic restraint stress (Hunter et al., 2009). Reduced levels of H3K9me3 was detected, a finding that was reversed on administration of the antidepressant fluoxetine, though neither gene expression changes nor anxiety phenotype were measured (Hunter et al., 2009). Conversely, though decreased levels of H3K9me and H3K9me3 were also observed in young female rats exposed to early maternal separation, these changes were observed in the PFC, and underscored reduced fear startle (low anxiety phenotype; Kao et al., 2012). This suggests that though the same expression of histone marks are observed, these marks may govern different transcripts depending on the brain region. Additionally, sex differences should also be taken into consideration as it has been reported that maternal separation tends to illicit higher anxiety-like behaviors in male offspring compared with females (Lehmann et al., 1999).

Last, elevated levels of H3K9me3 associated with the GR promoter was detected in the amygdala and hippocampus of bHR rats (low anxiety) compared with their anxious bLR counterparts. This increase was concomitant with a decrease in GR expression, suggesting that H3K9me3 suppresses GR expression in these brain regions in a manner that attenuates HPA axis hyperactivity, a factor underscoring the anxiety response (Chaudhury et al., 2014).

Overall, these studies elucidate specific histone marks associated with unique gene expression patterns that underlie the anxiety models used. To deduce the upstream regulation of these histone marks, other studies have investigated the histone-modifying enzymes responsible for these trends.

#### Enzymatic regulation of histone modifications

The question that remains is as follows: what enzyme manages these observed H3K9 methylation states? The HMT complex G9a/G9a-like protein (GLP), composed of the HMT euchromatic histone-lysine *N*-methyltransferase 2 (EHMT2) or G9a and G9a-like protein (GLP or EHMT1), has been shown to be involved in the monomethylation and dimethylation of H3K9 (Schaefer et al., 2009). Researchers have reported that postnatal knockout of G9a reduced the anxiety phenotype in mice, while mutation or deletion of one copy of the GLP gene in humans leads to Kleefstra syndrome, characterized by social behavior impairment, impulsivity, aggression, and mental retardation (Schaefer et al., 2009). Thus, G9a/GLP seems to exert temporal effects on developmental histone methylation. In concordance with this hypothesis, researchers found that administration of UNC0642 or A-366, selective G9a/GLP inhibitors, had different anxiety outcomes depending on the age of drug reception. Embryos that were exposed to the drug *in utero* after pregnant dams received intraperitoneal injections of UNC0642 showed an increase in anxiety-like behaviors, while adult mice receiving either drug showed a significant reduction in the anxiety phenotype in a dose-dependent manner (Wang et al., 2018). Western blots conducted on whole-brain extracts of adult mice treated with either drug showed the expected decrease in H3K9me2, though adult mice exposed to UNC0642 during gestation showed no changes in the level of H3K9me2, suggesting that the effect of G9a/GLP is developmentally sensitive (Wang et al., 2018). The researchers believed that the observed reduction in anxiety in adult mice models make both UNC0642 and A-366 potential therapeutic options for treating anxiety. Though no specific brain region or specific gene such as BDNF, was investigated in this study, the correlation of reduced global H3K9me2 and the observed decrease in anxiety-like behaviors, aligns with the study by Suri et al. (2013), where an increase in repressive H3K9me2 led to decreased hippocampal neurogenesis and overall cognitive decline. This provides a potential therapeutic target—the G9a/GLP enzyme complex.

Suri et al. (2014) published a follow-up article to the maternal separation ES study that reported biphasic responses to an increase in hippocampal H3K9me2. While young adult rats showed an increase in BDNF expression, they also demonstrated high-anxiety behaviors in the open field test compared with controls. This was not observed in middle-aged ES and control animals (Suri et al., 2014). While BDNF upregulation appears to underlie improved spatial learning in young adult mice, it also seems to promote the development of the anxiety phenotype not observed in middle-aged ES animals. In addition to BDNF, young adult and middle-aged animals expressed distinct transcriptomes with very little overlap (Suri et al., 2014). Curiously, in young adult rats with anxiety-like behaviors, the genes *Grin1* and *Grik2*, which code for subunits of different glutamate receptors, were differentially regulated compared with controls of the same age. ChIP analysis of histone-modifying enzymes in these animals showed a biphasic expression of HDAC2 and HDAC8, such that young ES animals expressed reduced levels of these deacetylases compared with their middle-aged ES counterparts (Suri et al., 2013). Histone methyltransferase expression was also found to be altered, such that HMT Suv39h1 was downregulated in young adult ES rats and significantly upregulated in middle-aged ES rats. The differential expression of histone-modifying enzymes did not, however, translate into global acetylation and methylation changes in the brain, suggesting that differential anxiety-relevant histone modifications govern transcriptomes specific to each brain region (Suri et al., 2013). This follow-up study shows that multiple cooperating histone-modifying enzymes, such as HDACs and HMTs, modulate the anxiety transcriptome, though further investigation of gene clusters under the regulation of these enzymes would help to formulate a bigger picture.

#### Histone modifications associated with the HPA axis

Previous studies have shown that increased levels of corticosterone delivery to the CeA increases CRF mRNA, enhancing anxiety-like behavior as well as dysregulation of the HPA axis (Shepard et al., 2003). In a rat model, delivery of increased concentrations of CORTs was shown to induce chronic anxiety. Decreased H3K9ac was observed in animals infused with CORT compared with controls infused with vehicle on staining of CeA slices (Tran et al., 2015). Paralleling this finding, it has previously been reported that in a chronic variable stress rat model, H3K9ac and H4K12ac were decreased in the CA3 region of the hippocampus and DG (Ferland and Schrader, 2011). Though brain regions vary between studies, both studies suggest that a decrease in the H3K9ac marker is influenced by stressors and may underscore the anxiety phenotype.

H3K9ac has previously been shown to regulate the promoter of the glucocorticoid receptor (Zhang et al., 2013). This supports the finding that CORT-infused animals expressed lower levels of glucocorticoid receptors, because of the decrease in H3K9ac reported, with a 5–7 fold increase in Crf mRNA expression levels in the study by Tran et al. (2015). ChIP of H3K9ac revealed a significant decrease in acetylation at the GR promoter, emphasizing the role of H3K9ac as a permissive mark for GR expression. GR-ChIP-seq revealed that CORT administration reduced GR sequestering of transcription factor AP-1, an interaction that would have suppressed CRF expression via the negative feedback loop in the HPA axis. Instead, increased AP-1-mediated CRF expression is observed in CORT animals (Tran et al., 2015). Further investigation through antibody binding revealed colocalization of the HDAC sirtuin 6 (SIRT6) with GR, such that an increase in SIRT6 correlated with a decrease in GR expression. TSA, a previously mentioned HDAC inhibitor of class I and II HDACs, was found to significantly increase H3K9ac and GR expression, while decreasing CRF mRNA levels in the CeA. This was accompanied by rescue of the anxiety phenotype and a reduction in SIRT6 (Tran et al., 2015). The researchers summarize the mechanism as follows: increased CORT delivery activates GRs, which localize to the nucleus to suppress CRF production via AP-1 interaction. SIRT6 is recruited to the GR promoter, where it deacetylates H3K9, reducing GR expression. This alleviates the suppression of AP-1 by GR, and CRF expression is sustained at an increased rate (Tran et al., 2015). Studies such as these attempt to hone in on candidate therapeutic targets, such as SIRT6. In HAB mice bred for high anxiety, hypoacetylation of H3 was also observed in the cingulate cortex, adding to the growing literature suggesting that a decrease in histone acetylation marks in a variety of brain regions tend to underscore anxiety phenotypes. Treatment with the HDAC inhibitor MS-275 (Entinostat) was able to rescue these lower acetylation trends while exerting an anxiolytic effect (Sah et al., 2019). Overall, these studies suggest that there is a potential niche for HDAC inhibitors in the treatment of anxiety phenotypes (Sah et al., 2019).

#### Substance use comorbidity

ADs, such as GAD and social anxiety, are often comorbid with substance use disorders, such as abuse of cocaine and alcohol (Noyes, 2001; Buckner et al., 2008). Comorbidity incidence is often high because the same brain regions are implicated in both disorders, such as the nucleus accumbens (NAc). The NAc is the reward center of the brain that consists of many types of GABAergic neurons and is often implicated in cocaine use and addiction (Feng et al., 2014). Cocaine has been previously shown to induce a drug-specific transcriptome consisting of thousands of gene expression changes via differential histone modifications in the NAc (Feng et al., 2014). In an earlier study, severe downregulation of HDAC5 in the NAc was observed in mice exposed to chronic cocaine usage, as well as mice exposed to CSDS, via their own unique mechanisms (Renthal et al., 2007). Treatment of CSDS mice with the antidepressant imipramine, partially restored HDAC5 mRNA levels to near basal levels (Renthal et al., 2007). Furthermore, *Hdac5* knock-out mice developed more severe social avoidance behaviors following subjection to CSDS compared with wild-type counterparts (Renthal et al., 2007). Though the researchers investigated genes under the regulation of HDAC5 in cocaine-treated mice, they did not do so in CSDS mice (Renthal et al., 2007). It would be interesting to see whether there is any overlap in differential gene expression between these two models.

Another cocaine study conducted in rats showed that viral overexpression of G9a, one of the protein components of the HMT complex G9a/GLP previously discussed, resulted in an increase in H3K9me2 and was accompanied by an increase in rat sensitivity to cocaine and anxiety-like behavior (Anderson et al., 2018). These findings appear to align with those of the study by Wang et al., where a decrease in G9a was concomitant with a decrease in H3K9me2 in low-anxiety animals.

Recall that a decrease in H3K9ac in the CeA was observed in correlation with the anxiety phenotype in animals infused with CORT (Tran et al., 2015). In another study, P rats, bred for selective alcohol preference, have been shown to have higher *Hdac2* expression levels and a concomitant decrease in H3K9ac accompanied by anxiety-like behaviors (Moonat et al., 2013). Upon acute exposure to ethanol, P rats were found to have reduced expression levels of HDAC2, increased levels of H3K9ac at *Bdnf* exon IV promoter, and a diminished anxiety phenotype (Moonat et al., 2013). Selective inhibition of *Hdac2* by siRNA infusion also resulted in decreased HDAC2 mRNA and a concordant increase in H3K9ac, strengthening the supposition that HDAC2 modulates H3K9ac levels. An increase in dendritic spine density was observed in the CeA only, for both acute ethanol exposure and siRNA infusion (Moonat et al., 2013). Though the mechanism for HDAC2 inhibition by ethanol is unclear, the anxiolytic effect of alcohol in social situations can be inferred from this model, as it appears that alcohol consumption attenuates anxiety symptoms and phenotypes on an epigenetic level. In support of this hypothesis, P rats consumed significantly less alcohol on siRNA treatment and subsequent HDAC2 inhibition (Moonat et al., 2013).

Studies such as these emphasize that not only do epigenetic modifications regulate gene expression changes and have global CNS consequences that may render an individual susceptible to a multitude of psychiatric disorders, but that these differential changes may also sustain underlying psychopathologies in comorbid cases. The differential histone modifications discussed above are summarized in Table 5 below.

**Table 5:** Summary of differential histone modifications marks and histone modifying enzymes in stress/anxiety models

Marker	Sample retrieved	Stress model	Reference
↑H4ac ↑*BDNF* IV	Prefrontal cortex	Footshock conditioned male mice (10–12 weeks) with fear extinction learning (low anxiety)	Bredy et al., 2007
↓H3K9me2 ↑*BDNF* IV	Hippocampus	Male rats (P21 and 2 months) subjected to maternal separation	Suri et al., 2013
↑H3K9me2 ↓*BDNF* IV	Male rats (15 months) subjected to maternal separation		
↓H3K9me ↓H3K9me3	Hippocampus	Adult male rats (P70) subjected to CRS	Hunter et al., 2009
↓H3K9me ↓H3K9me3	Prefrontal cortex	Young female rats subjected to early maternal separation (low fear-conditioned startle)	Kao et al., 2012
↑H3K9me3 ↓GR	Amygdala; hippocampus	Adult bHR (low anxiety) rats	Chaudhury et al., 2014
↓G9a ↓H3K9me2	Whole brain	Adult mice treated with G9a inhibitors (low-anxiety phenotype)	Wang et al., 2018
↑G9a ↑H3K9me2	Nucleus accumbens	Viral G9a overexpression in male adult rats (high anxiety phenotype)	Anderson et al., 2018
*↓HDAC2 ↑HDAC3 ↓HDAC8 ↓Suv39 h1*	Hippocampus	Young adult rats (2 months) subjected to early maternal separation (anxiety phenotype)	Suri et al., 2013
↓H3K9ac ↓GR ↑*CRF* ↑SIRT6	Amygdala (central)	Male adult mice were infused with CORT (chronic anxiety)	Tran et al., 2015
↓H3K9ac ↓H4K12ac	Hippocampus	Adult male rats subjected to CVS	Ferland and Schrader, 2011
↓HDAC5	Nucleus accumbens	Mice subjected to CSDS (chronic anxiety)	Renthal et al., 2007
↑HDAC2 ↓H3K9ac	Amygdala (central)	Adult P rats bred for alcohol preference (anxiety phenotype)	Moonat et al., 2013

P, postnatal day.

### Pharmacological implications: possible epigenetic rescue by HDAC inhibitors

More recent anxiety therapies have focused on the use of HDAC inhibitors. For example, VPA, a mood stabilizer, has been shown to significantly increase H4 acetylation at *Bdnf* exon IV promoter as well as its mRNA in the PFC of fear-conditioned mice, when administered before extinction training (Bredy et al., 2007). These findings accompany the observation that VPA-treated mice that previously showed no fear extinction, had enhanced long-term memory that supported the extinction of conditioned fear (Bredy et al., 2007; Whittle et al., 2013). VPA has also been shown to restore previously reduced levels of H3K9 methylation, suggesting that the expression of histone-methylating enzymes may be regulated by specific histone acetylation marks (Kao et al., 2012). The administration of VPA before extinction training suggests that VPA or VPA derivatives may be beneficial pharmacological interventions that can be used alongside CBT to improve learning outcomes of new, adaptive behaviors (Gavin et al., 2011) as VPA may encourage synaptic plasticity through enhanced *BDNF* expression (Bredy et al., 2007).

Other HDAC inhibitors that have been shown to restore histone acetylation while rescuing from the anxiety phenotype in animals include Trichostatin A (Tran et al., 2015), Vorinostat (Fujita et al., 2012), and MS-275 (Sah et al., 2019). MS-275 did not rescue from deficient extinction acquisition in an S1 mouse model that showed no extinction learning (Whittle et al., 2013), while Vorinostat appeared to increase the expression of *Nr2b* (NMDA receptor gene) in the hippocampus via increased H3 and H4 acetylation at its promoter, a mechanism believed to facilitate fear extinction in rats (Fujita et al., 2012). This suggests that not only do HDAC inhibitors have varying degrees and mechanisms of effectiveness, but that the time of administration, such as before or during the application of a stressor versus after fear learning when the stressor has been removed, is crucial for its efficacy (Gavin et al., 2011).

## Summary of findings

In summary, significant differential DNA methylation and histone modifications have been reported between anxiety and healthy control subjects, impacting a plethora of genes, the abbreviations of which are listed in Table 1. Many studies suggest that exposure to a number of stressors *in utero*, such as maternal hormones released during stress (Xu et al., 2014; Kertes et al., 2016; St-Cyr et al., 2017), depression or anxiety (Vangeel et al., 2017; Zhu et al., 2018), endocrine disruptors (Zhou et al., 2013), low oxygen levels (Wang et al., 2013), as well as medications, may alter DNA methylation patterns in offspring, rendering them either resilient or vulnerable to developing an AD.

As established, DNA methylation is modulated by enzymes known as DNMTs (Morris et al., 2016). Decreased expression of *Dnmt3a* (Murphy et al., 2015; Elliott et al., 2016) and *Dnmt3b* (Elliott et al., 2010; Wang et al., 2013; Ishii et al., 2014) has been reported on multiple occasions in both anxious human and animal models, as well as in both peripheral and CNS tissue samples. In particular, reduced expression of *Dnmt3b* appears to accompany *Crh*/*Crf* expression increases observed in HPA axis hyperactivity and the anxiety phenotype, suggesting that Dnmt3b is responsible for suppressing *Crh*/*Crf* via repressive methylation at its promoter region (Elliott et al., 2010; Wang et al., 2013). Likewise, overexpression of the CRF receptor CRHR has also been reported on numerous occasions in anxiety models, further emphasizing that aberrant HPA axis regulation underscores this phenotype (Wang et al., 2013; Sotnikov et al., 2014). Increased methylation of *Nr3c1*, the glucocorticoid receptor gene, has been reported more often than not, in anxiety models (van der Knaap et al., 2014; Wang et al., 2017).

Decrease in GR expression may damper the negative feedback mechanism in HPA axis function, leading to hyperactivity and the observed anxiety phenotype (Tran et al., 2015; Wang et al., 2017; Schmidt et al., 2019). The histone mark H3K9ac has also been observed in a chronic anxiety model, such that a decrease in H3K9ac resulted in decreased GR expression and increased CRF production (Tran et al., 2015).

Differential methylation trends of GABA- and glutamate-associated genes have also been discussed. Although upregulation or downregulation of these genes has variable impacts on the anxiety phenotype depending on the age of the animals, the brain regions involved, and the type of stress used to induce the phenotype. Notably, DNMT1 appears to regulate GABA production through *Gad67* expression, such that an increase in DNMT1 is concomitant with decreased GAD67 transcription, possibly through increased methylation. A subsequent decrease in GABA is observed (Zhou et al., 2013; Zhu et al., 2018).

As it is most crucial to brain function and psychiatric disorders, *Bdnf* expression has been studied in detail. This gene provides a great model for illustrating the principle that epigenetic modifications are intricately tied together, such that a variety of markers cooperate to either silence or activate genes. In other words, no single epigenetic alteration acts independently. Methylation marks on the *Bdnf* promoter are bound by MeCP2, a methyl CpG binding protein that appears to recruit HDACs (Martinowich et al., 2003). These HDACs, particularly HDAC2 (Moonat et al., 2013), appear to silence *Bdnf* expression by removing permissive acetyl marks on associated histones such as H3K9ac, rendering the chromatin less available for active transcription of BDNF. As a result, reduced *Bdnf* expression can be rescued with the administration of HDAC inhibitors (Bredy et al., 2007), as well as acute ethanol exposure (Moonat et al., 2013), a finding that possibly explains the anxiolytic effects of alcohol consumption. Increased H4 acetylation has also been reported with concomitant *Bdnf* IV expression (Bredy et al., 2007). Histone acetylation marks are not the only histone modifications found to be associated with BDNF, however. A decrease in repressive H3K9me2 at the promoter of *Bdnf* exon IV has also been associated with increased *Bdnf* expression, and an increase in H3K9me2 has been shown to decrease this expression, highlighting a direct inverse relationship between the mark and *Bdnf* expression (Suri et al., 2013). Additionally, the histone methyltransferase G9a/GLP appears to be responsible for the presence of H3K9me2 such that an increase in G9a is concomitant with an increase in H3K9me2 and a decrease in G9a/GLP is observed when decreased levels of H3K9me2 are detected (Anderson et al., 2018; Wang et al., 2018). Decreased levels of G9a and H3K9me2 have been associated with low-anxiety phenotypes (Anderson et al., 2018). It is possible that this decrease in G9a/GLP and a subsequent decrease in H3K9me2 permit *Bdnf* expression that may account for the increased *Bdnf* expression trends observed in reduced anxiety phenotypes. It is vital that one should not assume that an increase in *Bdnf* expression will decrease anxiety phenotypes, as, depending on the age of the organism and the brain region undergoing BDNF-facilitated neurogenesis, BDNF may promote positive behavior learning such as fear extinction later in life or negative memory-associated learning, such as fear conditioning at younger ages.

Other implicated genes in anxiety studies include *Fkbp5* (Hartmann et al., 2015; St-Cyr et al., 2017; Schmidt et al., 2019) and the histone-modifying enzymes HDAC5 (Renthal et al., 2007), HDAC8, and the HMT Suv39h1 (Suri et al., 2013). Again, it is of great importance to understand that other epigenetic factors underlie AD pathology via cross talk (Schiele et al., 2020). ATP-dependent chromatin remodeling factors, such as SNF2H and CHD3, have been shown to be differentially expressed in the amygdala and ventral hippocampus, respectively, of high-anxiety mice (Wille et al., 2016). These chromatin remodeling factors, such as CHD3, are able to form NuRD complexes—large protein remodeling complexes—with HDAC1/2 (Wille et al., 2016). This mechanism highlights the notion that no single epigenetic marker works independently; rather, they work in concert with other chromatin remodeling factors to alter chromatin states and to modulate gene expression (Wille et al., 2016).

## Limitations and Future Directions

Though there is a substantial amount of literature investigating specific DNA methylation and histone modification trends underlying the anxiety phenotype, consensus is rarely unanimous or straightforward because of several factors.

1. While studies conducted in humans tend to use blood and saliva samples, peripheral fluids do not accurately represent brain neurochemistry involved in AD as each tissue type carries its own unique pattern of epigenetics (Braun et al., 2019). However, longitudinal studies in neonatal participants of cord blood methylation could elucidate potential markers for risk or resilience towards developing an AD or other psychiatric conditions in later life. Additionally, postmortem studies using human brain samples of known AD patients would add diversity to the epigenetic biomarkers currently reported. Likewise, the development of investigative tools like BECon, which aim to interpret blood-based DNA methylation findings within the context of the brain, could encourage more human-focused psychiatric studies in the future (Edgar et al., 2017).
2. Though there are multiple methods for modeling anxiety in animals such as rodents, as well as testing and measuring anxiety-like behaviors in these models, directly relating findings to human psychopathology often runs the risk of oversimplification as psychiatric disorders are characteristic of human beings and our complex thought processes and emotionality. Use of primate models, such as the macaques in the study by Alisch et al. (2014), would be ideal as they are more closely related to humans.
3. The majority of anxiety studies tend to focus on either specific brain regions, omitting other relevant structures, or to pool all samples by using the whole brain. Each brain region carries its own pattern of epigenetic alterations depending on the nature of the stressor and timing of application. Focusing on a single brain region within a study falls short of constructing a bigger picture of brain pathways altered in anxiety, while global measurements are often biased, crediting overall epigenetic changes to the anxious phenotype without regard for local changes. Studies that aim to investigate multiple brain regions individually within a single model could help to construct a larger picture of the anxiety phenotype on an epigenetic level.
4. Likewise, while anxiety-based studies tend to focus on specific DNA methylation marks or histone modifications, it is important to not lose sight of the fact that these regulatory markers work in tandem to alter gene expression and are not independent of each other. Oftentimes, signaling cascades involving these modifications are not investigated. While future studies could focus on newly studied epigenetic modifications, like histone serotonylation and dopaminylation, or lesser investigated mechanisms within the field of anxiety, such as nucleosome positioning and microRNA regulation of protein-coding genes, more holistic studies that attempt to tie DNA methylation patterns and DNMT expression to histone-modifying enzyme expression and histone markers could lead to a better overview of the bigger picture underlying the anxiety phenotype.
5. Last, many studies conclude before elucidating target genes, specific methylation sites, and gene expression patterns under the regulation of these differential epigenetic modifications. It would be prudent for future studies to fill in the gaps of previous work.

## Conclusion

The development of an anxiety disorder, like other psychiatric diagnoses, is multifactorial, with both an individual’s genes and their environment contributing to disease pathology. This is reflected in incidences of comorbidity, such that individuals fitting the criteria for an AD diagnosis may also be diagnosed with a substance abuse disorder or a mood disorder, such as depression. This reality, coupled with the notion that each biological component underscoring the anxiety phenotype is complexly layered, means parsing out individual causes of anxiety-specific psychopathology remains a challenge.

This area of neuroepigenetic research aims to identify patterns of epigenetic modifications that have an overall impact on genes and corresponding protein products that play key roles in sustaining ADs. Thus, the research reviewed here not only strives to better understand the ongoing pathology of ADs by identifying corresponding patterns across studies, but also to provide insight into how to efficiently address these underlying aberrations to alleviate symptom severity and improve quality of life. Notably, rodent models have been a great resource for discerning the efficacy of anxiolytic and antidepressant medications on psychopathology-related behavior, and though differential epigenetic modifications vary between model types, these animals can be used to further elucidate the mechanisms through which these drugs act, for safer and better targeted treatment regimens in the future.

While the lack of consensus among DNMT levels across anxiety models make these enzymes a harder treatment target group, possible anxiety treatments include more specific HDAC inhibitors, since this class of drugs have been shown to encourage *BDNF* expression and neurogenesis, a phenomenon that may bolster CBT and fear memory extinction when treating ADs. The detection of key proteins such as FKBP5, which when downregulated showed rescue from the anxiety phenotype, also offer attractive avenues for future pharmacological interventions. Additionally, the development of programs or algorithms that can bridge the gap between data obtained from peripheral tissue samples as biomarkers for the information gathered from the CNS, would be beneficial to the entire field of psychiatry as a diagnostic tool that can be used to screen for populations at risk.

The field is moving in a positive direction, but it is imperative to continue with additional collaboration between laboratories and institutes, harmonization of human and animal studies, and bridging findings from past research.

## Synthesis

Reviewing Editor: Michael Michaelides, NIDA-NIH Decisions are customarily a result of the Reviewing Editor and the peer reviewers coming together and discussing their recommendations until a consensus is reached. When revisions are invited, a fact-based synthesis statement explaining their decision and outlining what is needed to prepare a revision will be listed below. The following reviewer(s) agreed to reveal their identity: Anand Gururajan, Craig Werner.

Reviewer 1

In this review, the authors have compiled and reviewed the animal and human literature to outline the putative role for a dysregulated epigenome in the pathophysiology of anxiety disorders. It is a well written manuscript with perhaps a bit too much introductory detail and the authors could significantly cut-back on some sections to focus on the topic at hand, making reference to other reviews instead.

For example sections A and B can be summarised and authors could point to reviews. Similarly, there are many excellent reviews on animal models for aspects of anxiety so there is really no need to spend 4-5 pages on this.

The manuscript should start, i believe, with section C which includes a brief description of the methods used to profile the epigenome. I also would encourage the authors to make reference to RNA modifications and RNA editing as other post-transcriptional regulators of gene expression.

Page 23 - I feel FKBP5 deserves its own section.

Conclusions - “...neurogenesis by BDNF and HPA axis...cell cascades.” I feel like this paragraph doesn’t quite belong here and suggests there is futility in therapeutic targeting of the epigenome. I think the conclusion could be improved to not so much focus on the specific methodological improvements (e.g. detection primers) that need to be made to the field but rather provide a future outlook, more generally.

Reviewer 2

The authors of “DNA methylation and histone modification differences underlying anxiety pathophysiology: an epigenetic review” took on the monumental task of attempting to synthesize the epigenetic literature related to anxiety pathophysiology. While this is an extraordinary effort, there are major concerns related to the effectiveness of this review due to the lack of conciseness and coherence that need to be addressed.

Major concerns:

1) Many sections lack concluding sentences that summarize the studies described.

2) Multiple sections contain information that would be more effectively conveyed if condensed.

3) The review covers a great deal of literature, but the referencing is not exhaustive.

4) The important links between various brain regions and the epigenetic modifications are often not described.

Other comments:

1) The title would benefit from rephrasing.

2) Abstract: The authors should discuss how brain regions and the epigenetic signatures are critical to the expression of anxiety-like phenotypes.

3) AD pathology: The Introduction nicely summarizes the multifactorial complexity associated with anxiety; however, it would be useful if it addressed the theme of the review (epigenetic modifications).

4) Neurological Anatomy & Physiology: Despite being well-written it is not coherent with the theme of the review (anxiety). The authors discuss the role of stress and plasticity but stress can lead to other pathologies, including depression, and not just anxiety. These sections should be focused.

5) Hippocampus: Please provide references adjacent to statements such as anatomical differences of hippocampal structure.

6) “Studies have shown that the IL 6 is responsible for suppressing fear responses by inhibiting CeA output.” Please provide references.

7) The authors describe in the DNA methylation section that the DNMTs have specific methylation patterns. Do these methylation patterns have implications in psychiatric disorders? This is an interesting section and a bit more information would improve comprehension for readers.

8) “Attempts at elucidating molecular changes in the human brain is made difficult by the major obstacle of obtaining sample tissues. Findings gleaned from post-mortem brains must be considered warily as undocumented confounding factors such as recreational drug use and non-prescribed medicine abuse may skew results, as well as any time elapsed following death before brain retrieval and preservation”..... Please provide appropriate references.

9) “Most epigenetic studies conducted in humans usually involve the retrieval of peripheral samples such as cord or systemic blood or saliva. As such, much of neuroepigenetic psychiatric research relies on the use of animal models, particularly, rodents”.... Please provide appropriate references.

10) “To measure anxiety-like behaviors in animals, researchers must first create an anxiety model by applying different stressors to the animal. There are also a variety of rodents available that have been bred to have lower or higher responses to stress, or with different innate anxiety temperaments, with relevant anxiety outcomes. It is important to understand that while these stress paradigms very rarely mimic those experienced by humans, they evoke a relevant anxiety phenotype in the animal model. Stressors typically include restraint in a cylindrical, perforated tube, application of an electric shock to the foot (0.5-2mA) for 1-2 seconds, maternal separation following birth, cage tilting, disrupted light-dark cycles and food deprivation, to list a few (Campos 241 et al., 2013). Several stress paradigms that permit anxiety modeling may be employed.” This paragraph requires more comprehensive referencing.

11) The authors should discuss other anxiety-like behaviors such as marble burying, novelty-induced hypophagia and elevated zero mazes. These behaviors are also important to understanding the complex pathophysiology of anxiety.

12) There is emerging evidence that histone modifications can be mediated through monoamines, such as dopamine and serotonin. The authors could consider adding this information and how such epigenetic modifications could regulate anxiety disorders to encourage future research in these directions.

13) It is important to consider sex differences in in these epigenetic modifications and how they can differentially regulate anxiety-like phenotypes.

14) Line 20: “...changes in plasticity” should just be “plasticity”

15) Line 55: Needs a citation.

16) Line 60: Needs a citation.

17) Line 282: Should “reduced anxiety-like behaviors” be “increased”?

18) Line 668 (and others): Glutamate receptor subunit genes are called “glutamate receptors”, which is incorrect.

19) Table 3 should be introduced in the Introduction because it’s a very useful resource to have during reading.

## Author Response

Response to Reviewers

Reviewer 1

In this review, the authors have compiled and reviewed the animal and human literature to outline the putative role for a dysregulated epigenome in the pathophysiology of anxiety disorders. It is a well written manuscript with perhaps a bit too much introductory detail and the authors could significantly cut-back on some sections to focus on the topic at hand, making reference to other reviews instead.

For example sections A and B can be summarized and authors could point to reviews. Similarly, there are many excellent reviews on animal models for aspects of anxiety so there is really no need to spend 4-5 pages on this.

The manuscript should start, I believe, with section C which includes a brief description of the methods used to profile the epigenome. I also would encourage the authors to make reference to RNA modifications and RNA editing as other post-transcriptional regulators of gene expression.

Thank you for your thoughtful suggestions. The introductory sections summarizing the anatomical and physiological aspects of anxiety disorders have been summarized in a more concise manner and placed after the Introduction of the epigenome and the two epigenetic topics we cover in this manuscript: DNA Methylation and Histone Modifications. Additionally, we have redistributed much of the anatomy and physiology sections throughout the findings and discussion sections as needed within the context of the studies reviewed for easier understanding when reading.

We have also chosen to summarize the animal models and tests used in anxiety research into tables for more concise reading.

Lastly, we have chosen to reference RNA modifications and other post-transcriptional regulators in our Future Directions section.

Page 23 - I feel FKBP5 deserves its own section.

After further research and deliberation, FKBP5 was included as part of the subheading (line 355) discussing NR3C1 instead of being granted its own section as there is not enough data available within the scope of anxiety-focused epigenetics studies to fully flesh out a section specifically discussing FKBP5, though additional sources have been provided.

Conclusions - “...neurogenesis by BDNF and HPA axis...cell cascades.” I feel like this paragraph doesn’t quite belong here and suggests there is futility in therapeutic targeting of the epigenome. I think the conclusion could be improved to not so much focus on the specific methodological improvements (e.g. detection primers) that need to be made to the field but rather provide a future outlook, more generally.

Thank you for the great feedback. We agree that the conclusion suggests futility within the field and have opted to improve the conclusion as suggested by providing a more general future outlook. The language has also been reconsidered so that the tone of the conclusion reads to be hopeful as opposed to ineffectual.

Reviewer 2

The authors of “DNA methylation and histone modification differences underlying anxiety pathophysiology: an epigenetic review” took on the monumental task of attempting to synthesize the epigenetic literature related to anxiety pathophysiology. While this is an extraordinary effort, there are major concerns related to the effectiveness of this review due to the lack of conciseness and coherence that need to be addressed.

We thank you for your comments and suggestions and have taken the time to address every concern below.

Major concerns:

1) Many sections lack concluding sentences that summarize the studies described.

Thank you for your criticism. We have reviewed and edited the subsections under the findings section and added more conclusive statements that summarize the main takeaway points for the studies included.

2) Multiple sections contain information that would be more effectively conveyed if condensed.

Thank you for this suggestion. Please refer to the Response 1 to Reviewer 1 to see how we chose to summarize a few sections in order to facilitate more concise reading.

3) The review covers a great deal of literature, but the referencing is not exhaustive.

We agree with the reviewer and have revised the manuscript to include better references throughout. For example, Bakulski et al., 2016 (line 168), Wochnik et al., 2005 (line 392), Noyes, 2001 (line 793), Braun et al., 2019 (line 934) to list a few. Continuous referencing of already used sources have been inserted as well and are all highlighted blue for easier reading.

4) The important links between various brain regions and the epigenetic modifications are often not described.

We thank the reviewer for pointing this out. The initial introductory sections reviewing brain anatomy and physiology were meant to be a resource for the reader to better understand why specific brain regions were chosen for investigation in these studies. However, after some deliberation, we have opted to disperse the information in these sections throughout the findings section for easier reading and to make the connection between the regions and epigenetic modifications more clear. For example, lines 96-99, lines 120-121, lines 134-136, to list a few.

Other comments:

1) The title would benefit from rephrasing.

The title has been rephrased to: The Epigenetics of Anxiety Pathophysiology: A DNA Methylation and Histone Modification Focused Review, to better encompass the goals of this review.

2) Abstract: The authors should discuss how brain regions and the epigenetic signatures are critical to the expression of anxiety-like phenotypes.

The abstract has been entirely reconstructed in consideration of this suggestion to better reflect brain regions and epigenetic marks within the scope of anxiety-like phenotypes discussed in this review (lines 1-21).

3) AD pathology: The Introduction nicely summarizes the multifactorial complexity associated with anxiety; however, it would be useful if it addressed the theme of the review (epigenetic modifications).

Originally, we addressed the anatomical, then physiological and biochemical factors underscoring AD pathology with the goal of narrowing down to the epigenetic root of ADs. However, after considering Reviewer 1’s suggestion to start the manuscript with an introduction to the epigenome and its role in anxiety and this suggestion to better address epigenetic modifications, we think we have better addressed the theme of the review in the introduction.

4) Neurological Anatomy & Physiology: Despite being well-written it is not coherent with the theme of the review (anxiety). The authors discuss the role of stress and plasticity but stress can lead to other pathologies, including depression, and not just anxiety. These sections should be focused.

Thank you for this feedback. This section, Neurological Anatomy & Physiology, has been removed and redistributed within the context of the anxiety-focused studies discussed in the result section of this manuscript so as to better provide a more comprehensive understanding of brain anatomy involved in AD pathology.

5) Hippocampus: Please provide references adjacent to statements such as anatomical differences of hippocampal structure.

This section has been entirely removed in response to previous reviewer comments.

6) “Studies have shown that the IL 6 is responsible for suppressing fear responses by inhibiting CeA output.” Please provide references.

This statement was edited out upon revision.

7) The authors describe in the DNA methylation section that the DNMTs have specific methylation patterns. Do these methylation patterns have implications in psychiatric disorders? This is an interesting section and a bit more information would improve comprehension for readers.

Methylation patterns of DNMTs were mentioned within a background context with no relevance to the anxiety studies discussed. We have removed this statement as these patterns have not been specifically studied within the framework of anxiety.

8) “Attempts at elucidating molecular changes in the human brain is made difficult by the major obstacle of obtaining sample tissues. Findings gleaned from post-mortem brains must be considered warily as undocumented confounding factors such as recreational drug use and non-prescribed medicine abuse may skew results, as well as any time elapsed following death before brain retrieval and preservation”..... Please provide appropriate references.

References provided, line 172.

9) “Most epigenetic studies conducted in humans usually involve the retrieval of peripheral samples such as cord or systemic blood or saliva. As such, much of neuroepigenetic psychiatric research relies on the use of animal models, particularly, rodents”.... Please provide appropriate references.

References provided, lines 173-174.

10) “To measure anxiety-like behaviors in animals, researchers must first create an anxiety model by applying different stressors to the animal. There are also a variety of rodents available that have been bred to have lower or higher responses to stress, or with different innate anxiety temperaments, with relevant anxiety outcomes. It is important to understand that while these stress paradigms very rarely mimic those experienced by humans, they evoke a relevant anxiety phenotype in the animal model. Stressors typically include restraint in a cylindrical, perforated tube, application of an electric shock to the foot (0.5-2mA) for 1-2 seconds, maternal separation following birth, cage tilting, disrupted light-dark cycles and food deprivation, to list a few (Campos 241 et al., 2013). Several stress paradigms that permit anxiety modeling may be employed.” This paragraph requires more comprehensive referencing.

References provided, line 196.

11) The authors should discuss other anxiety-like behaviors such as marble burying, novelty-induced hypophagia and elevated zero mazes. These behaviors are also important to understanding the complex pathophysiology of anxiety.

We are grateful for this feedback, however, given the length of this manuscript, we have opted to only mention these assays in passing as they are not utilized by the investigators in any of the studies discussed here, and are therefore, do not directly pertain to the goals of this paper.

12) There is emerging evidence that histone modifications can be mediated through monoamines, such as dopamine and serotonin. The authors could consider adding this information and how such epigenetic modifications could regulate anxiety disorders to encourage future research in these directions.

This is a great suggestion. Though further research did not present any anxiety-focused studies that investigated other types of histone modifications aside from methylation and acetylation, we have briefly mentioned that other modifications, such as dopaminylation and serotonylation, provide future avenues to be explored, line 964.

13) It is important to consider sex differences in in these epigenetic modifications and how they can differentially regulate anxiety-like phenotypes.

While we agree that it is important to consider sex differences in the modifications discussed and how these underscore the anxiety phenotype, only one of these studies, Wang et al., 2013, suggested sex differences in the modifications or gene expression looked at. We acknowledge that majority of the studies discussed were single-sex studies, another limitation encountered within the field.

14) Line 20: “...changes in plasticity” should just be “plasticity”

”...changes in plasticity of various...” edited to “changes in various”

15) Line 55: Needs a citation.

Citation added, now line 121.

16) Line 60: Needs a citation.

Citation added, now line 331.

17) Line 282: Should “reduced anxiety-like behaviors” be “increased”?

While the original wording may have been confusing, no, rodents displaying increased anxiety-like behaviors would hide in the dark side of the box for longer periods of time instead of exploring and staying in the lighted side of the box, where they are exposed and vulnerable. This has been reworded more clearly in Table 2.

18) Line 668 (and others): Glutamate receptor subunit genes are called “glutamate receptors”, which is incorrect.

Thank you. We have rephrased this statement to say “...genes which code for subunits of different glutamate receptors...”

19) Table 3 should be introduced in the Introduction because it’s a very useful resource to have during reading.

Thank you for this suggestion. We have moved this table to the beginning of the Findings Section to provide a better resource when reading.
